# Temporal tracking of quantum-dot apatite across in vitro mycorrhizal networks shows how host demand can influence fungal nutrient transfer strategies

**DOI:** 10.1038/s41396-020-00786-w

**Published:** 2020-09-28

**Authors:** Anouk van’t Padje, Loreto Oyarte Galvez, Malin Klein, Mark A. Hink, Marten Postma, Thomas Shimizu, E. Toby Kiers

**Affiliations:** 1 Laboratory of Genetics, Wageningen University & Research, Droevendaalsesteeg 1, 6708 PB Wageningen, The Netherlands; 2 Department of Ecological Sciences, Faculty of Earth and Life Sciences, Vrije Universiteit Amsterdam, de Boelelaan 1085, 1081 HV Amsterdam, The Netherlands; 3 AMOLF Institute, Science Park 104, 1098 XG Amsterdam, The Netherlands; 4 Section of Molecular Cytology, van Leeuwenhoek Centre for Advanced Microscopy, Faculty of Science, University of Amsterdam, Science park 904, 1090 GE Amsterdam, The Netherlands

**Keywords:** Microbial ecology, Plant ecology, Evolution, Fungi

## Abstract

Arbuscular mycorrhizal fungi function as conduits for underground nutrient transport. While the fungal partner is dependent on the plant host for its carbon (C) needs, the amount of nutrients that the fungus allocates to hosts can vary with context. Because fungal allocation patterns to hosts can change over time, they have historically been difficult to quantify accurately. We developed a technique to tag rock phosphorus (P) apatite with fluorescent quantum-dot (QD) nanoparticles of three different colors, allowing us to study nutrient transfer in an in vitro fungal network formed between two host roots of different ages and different P demands over a 3-week period. Using confocal microscopy and raster image correlation spectroscopy, we could distinguish between P transfer from the hyphae to the roots and P retention in the hyphae. By tracking QD-apatite from its point of origin, we found that the P demands of the younger root influenced both: (1) how the fungus distributed nutrients among different root hosts and (2) the storage patterns in the fungus itself. Our work highlights that fungal trade strategies are highly dynamic over time to local conditions, and stresses the need for precise measurements of symbiotic nutrient transfer across both space and time.

## Introduction

Underground, arbuscular mycorrhizal fungi form massive physical networks of hyphae connecting roots of diverse host plants [1]. A single gram of soil can contain ten to hundreds of meters of hyphae that function as a conduit for nutrient transport [2, 3]. These networks, also called common mycorrhizal networks (CMNs), have tremendous effects on nutrient cycling, transferring up to five billion tons of carbon (C) per year worldwide [4–6]. Partnerships with mycorrhizal-type fungi have facilitated major evolutionary events across the globe [7, 8], driving the evolution of complex root traits [9, 10], and creating habitats for various other organisms [11, 12].

Past work has shown that mycorrhizal fungi mediate the success of their hosts, influencing which plants survive and reproduce [13–15]. The fungus does this by forming an intricate hyphal network, which explores the soil and gains access to soil-bound mineral nutrients, such as phosphorus (P) and nitrogen (N). The fungus then exchanges these nutrients for C compounds from the host plant [16–18]. Transfer of resources from a fungus to the host has been shown to be highly variable, and depend on factors including available soil nutrients [19, 20], host species [21], host age [22], light availability [23–27], and even host sex [28]. Because mycorrhizal networks can connect several plants simultaneously, the transfer of resource across a shared fungal network can shift depending on these factors. Pioneering work has shown how changing the environment [29] or host composition [30, 31] across a shared network will change resource allocation by the fungus. However, the factors determining exactly which plants get what resources remains an open question.

An emerging body of research is now asking whether we can predict fungal trading strategies across networks containing multiple host plants with diverse demands [32–34]. We define trading strategy as a “conditional strategy that prescribes trading behavior under all circumstances regularly encountered by members of the trading agent’s species” [35]. Trading strategies of noncognitive agents, such as plants and fungi, are assumed to be shaped exclusively by natural selection. Understanding these trading strategies is important if we hope to manipulate rates of nutrient transfer from the fungus to the host in agricultural settings [36]. For example, it is unknown how the host’s nutritional needs influence the amount and timing of resource transfer by the fungus. When connected in a multiple plant network, do arbuscular mycorrhizal fungi provide more nutrients to plants with higher nutrient demands? And if yes, is there a clear fitness benefit to fungi providing more nutrients to more nutrient demanding hosts?

Until recently, we have been limited in our ability to study the relationship between host demand and fungal trading strategies across shared fungal networks because we have been unable to effectively track the temporal dynamics of the nutrient transfer in the arbuscular mycorrhizal symbiosis. Understanding temporal dynamics is important because fungal strategies are likely to be transient, such that trade with one host, in time, does not imply a consistent supply of nutrients to the same host over an extended period of time [37]. We recently developed a technique to resolve this constraint that allows us to follow P resources tagged with differently colored nanoparticles from a fungal network to host roots [38]. We tagged apatite, a form of mineral P, with quantum dots (QDs). QDs are nanoparticles that fluoresce bright and pure colors when excited with UV light. We used a class of QDs that were highly fluorescent, stable and can fluoresce in different colors depending on chemical composition of the core of the QD [39, 40]. Each color has the same size and weight, making them physically indistinguishable [38]. The outer layer of carboxyl polymers protects the organisms from the heavy metal core, and allowed us to conjugate the QDs to P. To be able to study transfer strategies over time, we synthesized three colors of fluorescent P that were added in a time series. Using confocal microscopy and epifluorescence analyses on plant tissue, we could study where and when P was transferred to different hosts over time, and/or stored by the fungal network.

**Fig. 1:**
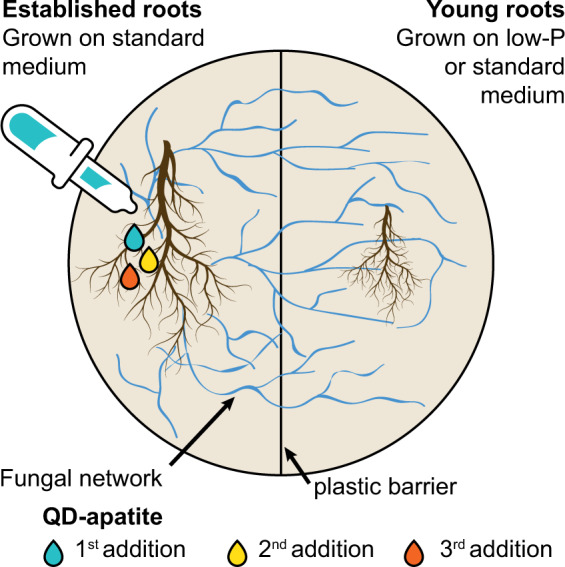
Experimental design. We grew two in vitro carrot roots on a split Petri dish, separated by a physical plastic barrier. The established roots grew on standard MSR and young roots grown on either standard MSR or a low-P MSR. The fungal hyphae crossed the barrier and formed a common mycorrhizal network between the two root systems. We added QD-apatite of three colors into the established root compartment over 3 weeks: first injection, 21 days before harvest (green), second injection, 14 days before harvest (yellow), and the third injection, 7 days before harvest (red).

Our aim was to understand what determines the temporal dynamics of when, and how much, P is transferred to individual root hosts connected by a single mycorrhizal network. Specifically, we asked: (1) does the mycorrhizal network transfer more P to host roots with a higher nutrient demand? (2) Do patterns of P allocation change over time? (3) Is there evidence that the fungus benefits from transferring P to host roots with the highest need? To answer these questions, we designed an in vitro root organ experiment in which a single fungal network colonized two physically separated transformed carrot roots of different ages, of which one was more P limited than the other (Fig. 1). We used a double plate system to physically isolate the roots from each other in separate compartments, while maintaining a living fungal connection between them. The older roots were grown for 6 weeks before the younger roots were transferred into the network. We added QD-tagged apatite at three time points in the compartment of the established root, each a different color, so we could quantify P transfer from the network to the individual roots by destructively harvesting them across time. Ultimately, we were interested in what mediates transfer from the fungal network to different hosts, and whether these trading strategies change over time.

## Materials and methods

### Cultures and growing conditions

We grew in vitro Ri T-DNA transformed carrot (*Daucus carota*) root organ cultures colonized with the fungus *Rhizophagus irregularis* (strain A5 Sanders Lab, University of Lausanne, Switzerland). Each root was grown in an individual compartment of a two compartment, 9-cm Petri dish. The compartments were separated by a plastic barrier that the fungal hyphae could cross, but the roots could not. The plastic barrier also prevented diffusion of nutrients across the plate, ensuring that all movement of QD-apatite was limited to transfer via the network [38]. This created an experimental setup with two root systems connected via a single fungus (Fig. 1). In the low-P treatment, one root compartment was filled with a low-P Modified Strullu–Romand (MSR) medium solidified with 4 g L^−1^ Phytagel (Sigma–Aldrich, St. Louis, MO, USA), containing only 1% of the amount of P (2.287 µmol P/L medium, low-P treatment), and the other root compartment was filled with standard MSR medium (228.7 µmol P/L medium) [41]. In the control treatment, both compartments were filled with standard MSR medium. For both treatments, we grew 32 replicates, and all replicates established a shared fungal network between the host roots. Five plates of the low-P treatment were discarded due to contamination.

At the start of the experiment, we transferred a branching root segment of 2 cm (~8.8 mg wet weight) to the root compartment with standard MSR. This was inoculated with a 1.5 × 1.5 cm^2^ block of fungal inoculum containing ~400 spores, hyphae, and colonized roots of 3 months old [42]. We allowed the symbiosis to establish on a single root system (called “established root”). After 6 weeks, we transferred a second root segment of 2 cm (~8.8 mg wet weight) to the other side of the Petri dish. This second root was grown on either standard MSR (control treatment) or low-P MSR medium (low-P treatment), and called a “young root” referring to the 6-week age difference between when the roots were transplanted into the Petri dish from a starting culture. The double compartment system was used so that we could add QD-apatite to one root compartment and determine how much was transferred through the fungal network to the second root system. We sealed the Petri dishes with a double layer of Parafilm M (Bemis Company, Inc., Neenah, WI, USA) and stored the Petri dishes in the dark at 25 °C, and at an angle of 45° as an additional measure to keep the established roots down and prevent them from crossing to the young root compartment.

### QD-tagged apatite

We constructed three differently colored QD-apatite solutions to track P transfer strategies over time following the procedure of Whiteside et al. [38]. We tagged hydroxyapatite, a naturally occurring rock phosphate [43], with QDs. We created QD-apatite by adding 150 mg Carboxyl CdSeS/ZnS Nanocrystals (CrystalPlex, Pittsburgh, PA, USA) of each color to 1 L 50% modified simulated body fluid (SBF)–50% SBF solution (11.9919 g NaCl; 1.96577 g NaHCO_3_; 0.447 g KCl; 0.4574 g MgCl_2_6H_2_0; 0.261 g K_2_HPO_4_; 0.4162 g CaCl_2_; 0.1062 g Na_2_SO_4_) [44, 45]. To conjugate the hydroxyapatite with the QDs, we performed two reactions in the dark. First, we formed small (~8 nm) P crystals by placing the solutions for 24 h at 37 °C. We then placed the solution on a shaker (100 oscillations/minute) for 24 h at room temperature. For the second reaction, we returned the solutions to 37 °C for another 60 h, allowing the smaller crystals to conjugate to bigger crystals (~200 nm), which closely mimics apatite crystals found in nature [46]. To remove unbound reagents in the absence of centrifugation, we replaced 80% of the precipitant with nanopure H_2_O (Nanopure^TM^, Thermo Fisher Scientific, Waltham, MA, USA) twice. Between the two washing steps, we shook the solutions by hand to reprecipitate. Each nmol QD-apatite contained ~700 nmols of P (nmol P:QD = 708:1) [38]. Finally, we brought the P to a concentration of 228.7 nmol/mL by diluting with nanopore H_2_O. We autoclaved the solutions and stored them at 4 °C in the dark.

Ten days after the young roots were transferred to either low-P or control conditions, we pipetted 1 mL green (488 nm) QD-apatite solution directly in the center of the established root compartment. This timing was chosen so that the fungal network was active and that the network from the established root had time to colonize the young root. One week later, we pipetted yellow (572 nm) at the same location and another week later red (666 nm) QD-apatite. Seven days after the third and final QD-apatite addition, all plates were harvested. We confirmed that all transfer between plants was via the fungal network based on a series of control experiments in which no movement of QDs across the plastic divider was found in the absence of a living fungal network [38].

### Harvest

We destructively harvested all Petri dishes by placing them at −80 °C for a minimum of 24 h to stop all metabolic reactions. To separate the fungus, roots, and MSR, we placed the content of each compartment in individual 50 mL centrifuge tubes (Greiner Bio-One International GmbH, Kremsmünster, Austria). We dissolved the MSR by adding 25 mL 10 mM sodium citrate solution. After incubating for 2 h at 65 °C to allow any substrate on the outside of the hyphae to be washed away, we separated the extraradical hyphae from the roots and placed the roots in paper bags to dry for a minimum of 48 h at 50 °C. From the dissolved MSR, we took a 1 mL subsample for confocal microscopy analysis of the extraradical hyphal network. We vacuum filtered each sample over a 0.45 µm, 47 mm cellulose nitrate Whatman membrane filter (GE Healthcare, Chicago, USA) to isolate the extraradical hyphae, and then freeze dried the network for 24 h [42]. We recorded dry weight of extraradical hyphae and roots. We manually homogenized the roots and divided them into two subsamples. From both the established and young roots, we subsampled ~7 mg for epifluorescence analyses, confocal microscopy, light microscopy, and to determine the overall phosphorus concentration of the roots. We subsampled ~20 mg of the larger established roots, and ~1 mg of the young roots for DNA extraction because of their smaller size. We pulverized the root samples for epifluorescence and DNA extraction using glass beads and a bead-beater for 40 s on speed 4 (Thermo Savant FastPrep Fp120 Cell homogenizer, Thermo Fisher Scientific, Waltham, MA, USA).

### DNA isolation and real-time quantitative PCR (qPCR) analysis

We isolated fungal DNA from the roots for intraradical hyphal abundance using the DNeasy Plant Mini kit (Qiagen, Hombrechtikon, Switzerland), with the exception that after the lysis step we added 10 µL internal standard, a vector of the cassava mosaic virus, to control for DNA extraction efficiency [42]. To measure the intraradical hyphal abundance, we used a TaqMan probe-based qPCR (LightCycler CFX96, Bio-Rad, Hercules, CA, USA), with probes and primers to target the lesser subunit of mitochondrial DNA of *R. irregularis* [47]. We analyzed each sample on internal standard and *R. irregularis* abundances and exported Cq values at a baseline threshold of 500 relative fluorescent units. We used the internal standard abundance to control for DNA extraction efficiency [42, 47] and converted Cq values to *R. irregularis* mtDNA copy numbers per mg of host root in R version V.3.3.1 [48] with the calibration curves described as described in [47].

### Visual root colonization measures

We visually determined the percentage of hyphal colonization, arbuscules, and vesicles by staining a subsample of ~9 mg in a subset of the roots (five root samples per treatment) with trypan blue [49]. We rehydrated the roots in dH_2_O and added 2.5% KOH solution to the dried root samples (w/v) and mixed well. We incubated the roots for 5 min in a water bath at 90 °C. After 5 min of cooling, we removed the KOH and rinsed the roots on a sieve with dH_2_O. We added 2 mL of staining solution (0.05% trypan blue in destaining solution: lactic acid/glycerol/dH_2_O 2:1:1) and mixed well. We incubated the roots for another 10 min at 90 °C in a water bath and after 5 min of cooling outside the water bath, we removed the staining solution by rinsing the roots with dH_2_O on a sieve. We stored the roots in Eppendorf tubes in glycerol/dH_2_O (1:1) at 4 °C. We analyzed the roots by placing roots on a microscopic slide under an Olympus CX41 microscope with a ×10 ocular and ×40 objective. We scored intraradical hyphae, arbuscules, and vesicles at gridline intersections.

### Fluorescence analysis

We analyzed root samples for fluorescence intensity to measure QD-apatite concentrations of three spectra in the roots. We prepared the samples by adding borate buffer (10 mM at pH 7.4) per 1 mg dry root per 150 µL buffer. We then placed five times 1 mg root of each subsample into a 96-well glass bottomed plate (Eppendorf AG, Hamburg, Germany). We obtained spectra from each sample well in the wavelength range of *λ* = 450–800 nm with 2 nm intervals using a BioTek Synergy MX plate reader at 325 nm excitation (BioTek Instruments, Bad Friedrichshall, Germany). To prevent edge effects, we left the outermost wells empty. To convert photon count to QD concentrations of different colors, we made calibration curves from each color based on seven QD concentrations (13.1, 9.83, 7.37, 5.53, 4.15, 3.11, and 2.33 mM [38]).

We used Matlab R2016a (The MathWorks, Natick, MA, USA) and a custom designed script in Matlab Code based on emission finger printing to deconvolute overlapping emission spectra, quantifying the three colors of QD-apatite simultaneously as described in detail in [38]. This approach allowed us to analyze low levels of QD-apatite (>1 femto mol QD per mg plant tissue) that would otherwise be undetectable using traditional filter and channel-based techniques. We subtracted background emission, and unmixed the overlapping emission spectra with linear models using reference spectra for the autofluorescence and the three QD colors. After unmixing, we optimized and smoothed the spectrum curves to reduce noise and normalized the data. We summed the photon counts of each color separately and converted the total photon counts to nmol of QD-apatite using the above described calibration gradients. To avoid pseudoreplication, we averaged over the five wells.

### Total P content of roots

As a second metric to confirm that QD-apatite was being transferred as a P source to host roots, we determined total root P concentration via acid digestion and spectrophotometry. This total P content includes P present in the Phytagel substrate in which the roots grew. We digested a subsample of root material by adding 2 mL digestion mixture (HNO_3_/HCl 4:1) per root sample to a Teflon cylinder. After 15 min we closed the cylinders, and placed them for 7 h at 140 °C. We then added 8 mL dH_2_O to each cylinder and transferred the contents of the cylinder to a test tube. We left the test tubes for 1 day at room temperature after which we closed the tubes with plastic foil and placed them at 7 °C for 7 days. We then determined the total P content with spectrophotometry. We added 4 mL reagent (1000 mL H_2_O, 13.33 mL H_2_SO_4_, 1.14 g (NH_4_)_6_Mo_7_O_24_·4H_2_O, 1.00 g C_6_H_8_O_6_, 2.6 mg K) to 150 µL of each root sample. After 30 min, we measured the absorbance at 880 nm UV–visible spectrophotometer UV-1601PC (Shimadzu, Kyoto, Japan) and calculated the concentration of P per mg sample. Total nmol of P per mg of root was then used as the denominator to calculate the % of P in the host root derived from QD-apatite, based on the ratio (QD-apatite:P = 1:708) as determined previously [38].

### Raster image correlation spectroscopy (RICS) analysis

Epifluorescence analysis on root tissue cannot distinguish between QD-apatite in the root cells and in the intraradical hyphae. Therefore, we used RICS, a technique that can measure particle concentrations from confocal images, to quantify the amount of QD-apatite in root cells versus the amount retained in fungal hyphae [50, 51]. We randomly selected five replicates per treatment and placed these on a microscope slide with 250 µL bicarbonate buffer. We analyzed all samples using an Olympus FluoView™ FV1000 confocal microscope (Olympus, Tokyo, Japan) with a water immersed ×60 UPLSAPO, NA 1.2 objective (Olympus, Tokyo, Japan). Excitation was conducted using a 20 MHz pulsed 405 nm laser (Picoquant, Berlin, Germany). Per sample, we collected five images of intraradical hyphae and five images of uncolonized root cells. The images we collected were 4.096 × 4.096 µm at a ×50 zoom, with an individual pixel sizes of 16 nm (256 × 256 pixel frames). We used an optimal pixel dwell time of 20 µs pixel^−1^ and a raster line time of 1.25 ms. With the SIM-FCS software [51], we calculated the RICS autocorrelation function, fitted with a 3D diffusion model, and corrected with the moving average intensity correction. We then could calculate the number of particles in nmol per bio-volume root [50, 51].

### Growth rate of roots

In order to determine growth rates of roots in our confined system, and confirm that roots were not limited by carbon over the time frame of our experiment, we imaged the growth of Ri T-DNA transformed carrot (*D. carota*) root organ cultures in a separate experiment. We imaged non-colonized and roots colonized with *R. irregularis* for 5 months. We filled standard 9-cm Petri dishes with MSR medium, and placed a young piece of ~2 cm root on top. We inoculated the root by positioning a circular agar plug, containing *R. irregularis* spores and hyphae on top of the roots. To provide similar conditions between the inoculated and non-inoculated plates, we added a sterile and geometrically identical agar plug to the non-colonized roots. We closed the Petri dishes with a double layer of Parafilm M and placed the Petri dishes in incubator at 25 °C in complete darkness.

The imaging system was setup in the incubator. It consisted of a rotary mount, allowing us to image up to six plates automatically. The plates were illuminated exclusively during each capture using dark-field illumination. A single picture per plate was taken with a DSLR camera (EOS 1100D Canon, Tokyo, Japan) placed on top. We imaged the Petri dishes every 2 h for 5 months. The growth rate of the roots was calculated by binarizing the images, in which pixels containing root are one, and the others are zero. The fraction of the gel surface area covered by root was calculated by summing the pixels in the binary image, and dividing by the total number of pixels in the imaged gel area at every time point (Fig. S1).

### Videos of nutrient flows in fungal hyphae with and without QD-apatite

To visually confirm that QD-apatite had been uptake by the fungal hyphae, we recorded changes in the flow inside the hyphae with and without the addition of QD-apatite, monitoring for changes due to large particles. We prepared plates of Ri T-DNA transformed carrot (*D. carota*) root organ cultures colonized with *R. irregularis*, as above. When the extraradical network was formed (~45 days), 100 µL of QD-apatite solution was added to half the plates. Seven days later, we captured videos of the cytoplasmic flow in the hyphae in the treatments with and without QD-apatite. Videos were made using an inverted microscope (Nikon eclipse ti-E, Nikon, Tokyo, Japan) at ×100 magnification (CFI Plan Apo Lambda 100X Oil, Nikon, Tokyo, Japan). Bright-field illumination was used for plates without QD-apatite solution, while synchronous bright-field and fluorescence illumination was used when QD-apatite was also present. Plates were illuminated with an ultraviolet LED light source at 365 nm (CoolLED pE-4000). Grayscale videos of 1 min were recorded using a Hamamatsu Orca-Flash 4 camera at 100 fps with a resolution of 15 pixels per micrometer. For all videos, plates were opened and placed facedown to visualize the fungal hyphae directly.

### Statistical analysis

We performed all statistical analysis in R version 3.6.1 [48]. All data, scripts, and analysis are available at: https://github.com/anoukvantpadje/Two_roots. A summary of the data can be found in the Supplementary material (Table S1). We studied the effect of the treatment (control and low-P), compartment (established and young), and time (days since QD injection) on the amount of QD-apatite in the roots. We calculated the ratio of QD-apatite in young/established roots by dividing the amount of QD-apatite in young over the amount in established roots as pairs in a shared plate, and analyzed the effect of treatment and time. To analyze the location of QD-apatite in hyphae/roots, we used our RICS analysis. We calculated the ratio of QD-apatite present in the intraradical hyphae divided by the QD-apatite in the roots per biovolume, for both established and young roots and studied the effect of treatment, compartment and time. We calculated the intraradical fungal abundance per root by taking the logarithm of the total root weight multiplied by the *R. irregularis* copy numbers per gram of root, and studied the effect of treatment and compartment on the extra- and intraradical fungal abundance and the root weight.

We tested all data for normality with a Shapiro–Wilk test and for homogeneity of variances with a Levene’s test. We checked the distributions of the residuals by eye using QQ plots. Due to non-normality and heteroscedasticity, we transformed the QD-apatite in roots, the ratio of QD-apatite in young/established roots, the extraradical and intraradical fungal abundance by taking the square root. We transformed the location of QD-apatite in hyphae/root by taking the logarithm.

We analyzed QD-apatite in roots, the ratio of quantum-apatite in young/established roots, the extra- and intraradical fungal abundance and the root weight with linear mixed effect models, using the R package lme4 [52]. We assigned Petri dish as a random effect to correct for the nonindependent measurement of the two root systems growing on the same plate. We produced type II ANOVA tables with the Kenward–Roger method to the denominate degrees of freedom and F-statistics using the R Package lmerTest [53]. We also assessed the difference in the ratio of QD-apatite in young/established roots at specific time points with three separate Student’s *t* tests. We analyzed the location of QD-apatite in hyphae/roots with a linear model and produced type II ANOVA tables using the R Package car [54].

## Results

**Fig. 2:**
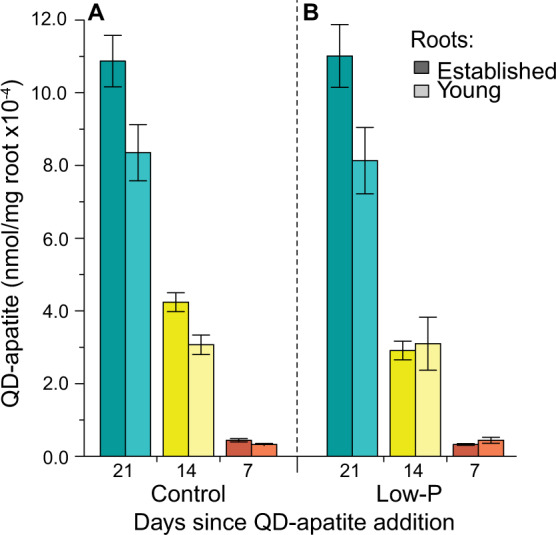
nmol QD-apatite per mg of host root. **a** Established roots in the control treatment contained significantly more QD-apatite per mg of root than young roots across all three time points: first addition, 21 days before harvest (green), second addition, 14 days before harvest (yellow), and the third addition, 7 days before harvest (red). **b** Established roots contained more QD-apatite from the first injection in the low-P treatment, but this significant difference disappeared by the last injection, 7 days before harvest (red) (*n*_control_ = 24, *n*_low-P_ = 25). Means ± SEM.

We first visually confirmed that QD-apatite was taken up by the fungal hyphae by comparing videos in which hyphae were exposed or not exposed to QD-apatite. We noted the large (~5 µm) vacuoles inside the hyphae when QD-apatite was present (Video S1), but never in the plates (ten replicates) when QD-apatite was absent (Video S2). We then quantified the distribution of QD-apatite across the established and young roots using epifluorescence measurements to determine how much was transferred by the mycorrhizal network from the established root compartment (where the QD-apatite was pipetted) to the younger roots. We found that QD-apatite per mg root (weight including intraradical fungal biomass) was significantly influenced by time, compartment (established versus young), and the interaction between time and treatment (Table 1). Specifically, in the first injection (i.e., green QDs, 21 days), we found more QD-apatite in the established roots compared to young roots, across both treatments (Fig. 2). However, this was a small difference given that the established roots had direct access to nutrients, while the young roots had access only indirectly via the fungal network. For example, in the control treatment, the young root had only 13% less QD-apatite per mg of root than the established, and in the low-P, the young root had only 15% < established root (Fig. 2).

**Table 1:** Analysis of variances.

	df	res	*F* value	*p* value
**QD-apatite per mg root**				
Time	2	222.439	1070.87	<0.0001*
Treatment	1	46.701	2.321	0.134
Compartment	1	229.886	18.751	<0.0001*
Time × treatment	2	222.462	3.424	0.034*
Time × compartment	2	222.468	2.891	0.0576
Treatment × compartment	1	230.33	2.611	0.108
Time × treatment × compartment	2	222.479	0.636	0.53
**Ratio of QD-apatite in young/established roots**				
Time	2	83.852	13.968	<0.0001*
Treatment	1	42.634	2.995	0.091
Time × treatment	2	84.123	1.838	0.165
**P per mg root**				
Treatment	1	58	11.478	0.001*
Compartment	1	58	1.382	0.245
Treatment × compartment	1	58	0.003	0.957
**%P from QD-apatite**				
Treatment	1	48	5.174	0.027*
Compartment	1	48	0.09	0.766
Treatment × compartment	1	48	0.771	0.381
**%P from 1st QD-apatite addition (21 days)**				
Treatment	1	48	7.066	0.011*
Compartment	1	48	0.127	0.723
Treatment × compartment	1	48	0.386	0.538
**%P from 2nd QD-apatite addition (14 days)**				
Treatment	1	48	0.08	0.778
Compartment	1	48	2.764	0.103
Treatment × compartment	1	48	1.301	0.26
**%P from 3rd QD-apatite addition (7 days)**				
Treatment	1	48	2.802	0.1
Compartment	1	48	6.812	0.012*
Treatment × compartment	1	48	6.245	0.016*
**QD-apatite retention**				
Treatment	1	42	5.419	0.025*
Compartment	1	42	4.676	0.036*
Time	1	42	3.129	0.054
Treatment × compartment	1	42	0.959	0.333
Treatment × time	2	42	5.421	0.008*
Compartment × time	2	42	1.352	0.27
Treatment × compartment × time	2	42	0.2	0.82
**Root biomass**				
Treatment	1	56.59	0.008	0.928
Compartment	1	56.204	846.59	<0.0001*
Treatment × compartment	1	56.59	0.783	0.38
**Extraradical hyphae biomass**				
Treatment	1	56.114	11.659	0.001*
Compartment	1	55.681	0.419	0.52
Treatment × compartment	1	56.114	0.003	0.954
**Intraradical colonization (copy numbers)**				
Treatment	1	52.496	5.318	0.025*
Compartment	1	37.803	305.721	<0.0001*
Treatment × compartment	1	52.496	0.653	0.423
**Percentage colonization**				
Treatment	1	103.918	1.894	0.202
Compartment	1	216.767	3.951	0.078
Treatment × compartment	1	34.191	0.623	0.45
**Percentage arbuscules**				
Treatment	1	0.8	0.0426	0.8411
Compartment	1	169.056	8.9945	0.0150*
Treatment × compartment	1	0.001	0	0.995
**Percentage vesicles**				
Treatment	1	1.6815	0.34	0.574
Compartment	1	31.142	6.302	0.033*
Treatment × compartment	1	2.9314	0.593	0.461

Degrees of freedom (df), residuals (res), *F* and *p* values are given for the effect of the variables. *p* values with a * have a significant effect of the variable (*p* < 0.05).

**Fig. 3:**
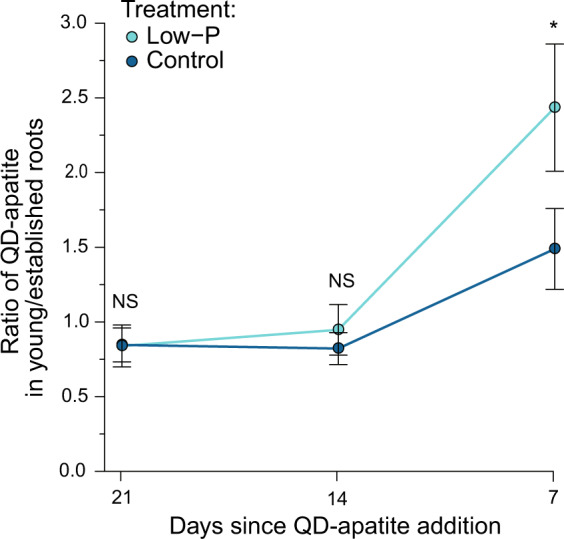
Ratio of QD-apatite in young/established roots. In the first two QD additions, there were no significant differences in allocation patterns of QD-apatite between young (light blue) and established roots (dark blue). However, by the third addition, the ratio was significantly higher, meaning more QD-apatite allocated to young roots, in the low-P treatment compared to the control (*n*_control_ = 24, *n*_low-P_ = 18). The asterisk indicates a *p* value < 0.05. Means ± SEM.

Second, we found that by the termination of the experiment, the nutrient condition for the host roots affected the nutrient allocation patterns of the fungus (Fig. 3). We plotted the ratio of QD-apatite in two host roots (young/established), allowing us to the study the transfer strategies of the fungus. We found a significant effect of time in both treatments (Table 1 and Fig. 3). We then compared the allocation patterns in each treatment for each time point. We found no statistically significant difference in allocation patterns of QD-apatite based on the first (Student’s *t* test: *t* = −0.067, *p* = 0.946) nor the second injection (Student’s *t* test: *t* = −0.506, *p* = 0.617). However, by the third injection, the ratio was significantly higher, such that there was more P allocated to young roots in the low-P treatment compared to the control (Student’s *t* test: *t* = −2.223, *p* = 0.032, Fig. 3). Over time, differences in nutrient transfer to roots could depend on P condition of the young root, with more nutrients being allocated to those roots in low-P conditions.

**Fig. 4:**
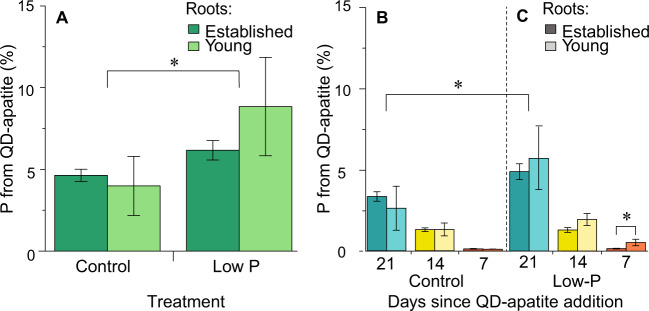
Percentage of P from QD-apatite in host roots. **a** Across all time points and treatments, an average of 5.6% of the P in the host roots originated from a QD-apatite source. The percentage of P from the QD-apatite was significantly higher in roots growing under low-P conditions. **b**, **c** In the first QD-apatite injection (21 days), %P from QD-apatite was significantly affected by treatment, with more P from QD-apatite in the low-P treatment. In the second QD-apatite injection (14 days), we found no significant effect of treatment, compartment, or the interaction. In the third QD-apatite injection (7 days), we found a significant effect of compartment and the interaction between treatment and compartment, with more P from QD-apatite in young roots in the low-P treatment (*n*_control,established_ = 32, *n*_control,young_ = 2, *n*_low-P,established_ = 24, *n*_low-P,young_ = 3). The asterisk indicates a *p* value < 0.05. Means ± SEM.

To confirm this trend using a different metric, we determined total P concentration of host roots via acid digestion technique (Fig. S2) and compared this to the % of P from fluorescing QD-apatite. We found that the % of P from the QD-apatite over total root P was significantly higher in roots growing under low-P conditions (~6.4%) compared to control (~4.7%) (Fig. 4a and Table 1). When plotting this as a time series, we found a significant effect of compartment (established versus young) and the interaction between treatment and compartment in by the third QD-apatite injection (7 days), with more P from QD-apatite in young roots in the low-P treatment (Fig. 4b, c and Table 1).

**Fig. 5:**
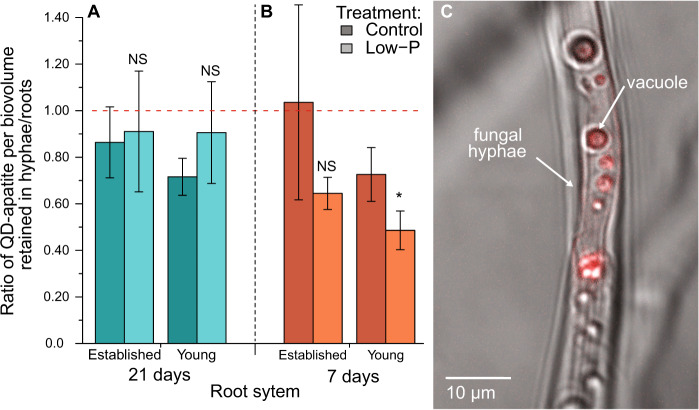
Retention of QD-apatite per biovolume in hyphae/roots for the first and last QD-apatite injection. We found that the spatial location of the QD-apatite was influenced by the treatment, compartment, and time of QD addition and by the interaction between treatment and time. **a** In the first addition (21 days), QD-apatite retention in hyphae was not significantly different between the established roots in the control (dark blue) or low-P treatment (light blue) nor between the young roots of the control or low-P treatment. **b** By the third addition (7 days), QD-apatite retention in hyphae was not different in the established roots of the control or low-P treatment. However, in the young roots, this ratio was lower in the low-P treatment than the control (*n*_control,established_ = 15, *n*_control,young_ = 12, *n*_low-P,established_ = 12, *n*_low-P,young_ = 15). The asterisk indicates a *p* value < 0.05. Means ± SEM. **c** Confocal image of a single hyphae with a vacuole containing QD-apatite.

Next, we were able to identify the exact spatial location of the QD-apatite (i.e., retained in the fungal hyphae versus transferred to the root) using RICS analysis. We found that spatial location of the QD-apatite was significantly influenced by treatment, compartment, and by the interaction between treatment and time (Table 1). From the first and second injection, we found no significant difference in the ratio of QD-apatite located in hyphae/roots between low-P versus control treatment. However, by the final injection, the ratio of QD-apatite in hyphae versus root was significantly lower in the young roots of the low-P treatment versus the control (Fig. 5 and Table 1). This result suggests that, rather than retaining the QD-apatite in the hyphae, the QD-apatite was more likely to be transferred to the roots, when there was a higher P limitation for roots. Over the time frame of our experiment, we found no significant effect of our treatment on root growth. Root biomass was not significantly different between the control and low-P treatments in both the established and young roots. We found that established roots were larger than young roots, as expected because they were planted 6 weeks earlier (Table 1). We then analyzed data from the root growth imaging experiment to confirm that roots were not C limited in the time frame of our experiment. We found root growth increased rapidly for the first 2 months, with root growth rate decreased to almost zero after ~3 months (Fig. S1a). Even after 3 months, we documented that the hyphal network continued to grow and sporulate (Fig. S1b), suggesting the allocation of C to the fungi was still not limited.

**Fig. 6:**
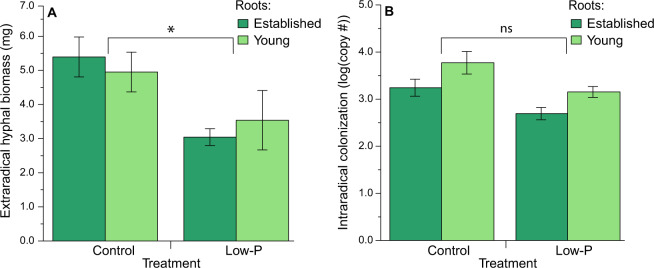
Fungal abundance. **a** Extraradical hyphal biomass was higher in the control treatment versus the low-P treatment, with no significant difference between the established roots (dark green) and the young roots (light green) in both treatments (*n*_control,established_ = 31, *n*_control,young_ = 32, *n*_low-P,established_ = 27, *n*_low-P,young_ = 23). **b** Intraradical colonization was higher in in control roots versus low-P roots, and younger roots had higher intraradical colonization per mg of root compared to the established host (*n*_control,established_ = 26, *n*_control,young_ = 17, *n*_low-P,established_ = 19, *n*_low-P,young_ = 4). The asterisk indicates a *p* value < 0.05. Means ± SEM.

We then studied the biomass of the fungi to determine if the way the fungus distributed nutrients among the two roots affected their own success. We asked whether providing more nutrients to the young root growing under the low-P conditions provided a benefit to the fungi, as measured by their extra and intraradical growth. We found overall higher biomass of extraradical hyphae in the control treatment compared to low-P, but no other statistically significant differences between young and established roots (Table 1 and Fig. 6a).

Finally, we analyzed intraradical fungal colonization per mg of root, as measured by *R. irregularis* copy number and visual colonization. We found a statistically significant effect of treatment and compartment on intraradical fungal colonization as measured by mtDNA copy number, but no effect in the interaction between treatment and compartment (Table 1). The colonization of control roots was on average 125 times higher than that of low-P roots. The colonization of the younger roots was on average 32 times higher than that of established roots (Fig. 6b). We visually quantified the % of hyphae, arbuscules, and vesicles using microscopy and found levels of hyphal colonization ranging from 18 to 38%, and arbuscules and vesicles around 0.5–10% of root length (Table 1 and Fig. S3).

## Discussion

Our aim was to understand the allocation dynamics of P transfer from fungal networks connected to two host roots using a recently developed QD-nutrient tagging approach. We asked (1) whether the distribution of P from a mycorrhizal network to a host root depends on the P needs of the host, specifically asking if fungal networks transfer more P to hosts growing under lower P conditions, (2) if fungal P allocation patterns changed over time, and (3) if the fungus benefited from providing more P to roots with a higher P demand.

### Nutrient need of the host plant influences the nutrient transport of fungi

First, our data suggest that QD-apatite can be taken up in one part of the fungal network and transferred through the network to a different host root. In our case, this movement was from an established root to a younger root (Fig. 2). We found that the P needs of the host can influence this distribution, however, these distribution differences take time to emerge. Specifically, we found that after 3 weeks, the fungus transferred more QD-apatite to young roots growing under low-P conditions (Fig. 2). By the third injection, the ratio of QD-apatite in young/established roots was significantly higher under low-P conditions than the control treatment (Fig. 3), and more of the total P was coming from QD-apatite (Fig. 4). Before this time point, we saw no effect of the nutrient status of the younger root. This small delay is not surprising given the nutrients were added directly to the compartment of the established roots, whereas the younger roots had access only through the shared network.

While we found that P allocation by the fungus is influenced by the P status of the host, the physiological mechanisms and regulating gene networks underlying this effect are still unknown. Key genes, such as *MtPT4* the mycorrhiza-inducible phosphate transporter, play an important role in regulating the P transfer at the symbiotic interface between the root cells and the fungus [55]. However, many additional (and complimentary) candidate genes in nutrient sensing and regulation are now emerging [56], including those involved in fatty acid transfer [57]. One major constraint in identifying underlying patterns in these regulation mechanisms is that similar physiological reactions can be driven by different molecular mechanisms in distantly related plant species [58].

Although we observed a higher amount of QD-apatite per mg of root in the established roots from the first injection, this was only 13–15% higher compared to younger roots (Fig. 2). This is a surprisingly small difference given that the established roots had direct access to the nutrients (including P readily available in the MSR medium), and thus these roots could potentially utilize a “direct pathway” via root transporters [59, 60], rather than relying on the fungus for P uptake and transfer. However, direct uptake of nutrients can be limited when the plants are colonized by arbuscular mycorrhizal fungi [60, 61, 62]. While direct uptake of QD-apatite by whole plants in the absence of mycorrhizal fungi has been documented, it was shown to be significantly less per mg of root compared to when mycorrhizal fungi are present [38]. This is because the rock phosphate apatite as a nutrient source is generally difficult for roots to solubilize.

Past work has shown that arbuscular mycorrhizal fungi can increase the dissolution and uptake of apatite [63, 64]. While the apatite uptake mechanism for arbuscular mycorrhizal fungi is still unknown, fungi generally use endocytic pathways to take up large particles [65–67]. For example, clathrin-mediated endocytosis has been shown in the fungus *Candida albicans*, with invagination cells reaching diameters of 100 nm [68]. Using bright-field and fluorescence imaging of flows, we found large vacuoles were formed inside the hyphae when the fungus was given access to QD-tagged apatite (Video S1). In hyphae not exposed to QD-tagged apatite, we noted the absence of those large vacuoles (Video S2). While endocytic pathways are likely to be responsible, we assume there is an initial dissolution of the apatite by mycorrhizal fungi prior to uptake. This process likely reduces the size of the tagged apatite crystals and facilitates translocation. There are many apatite solubilizing bacteria described [69, 70], but the ability of arbuscular mycorrhizal fungi to dissolute apatite in absence of any bacteria is still an active area of research. Previous studies have shown that mycorrhizal species *R. irregularis* and *Glomus custos* are able to dissolute apatite when in symbiosis with *Plantago lanceolata* in sterilized soil [63], and *Glomus clarum* increased the dry weight of maize plants fertilized with rock phosphate [71]. Likewise (although in non-sterile soil), the distantly related *Glomus tenuis* significantly increased P uptake from rock phosphate in four different crops [72]. In agreement with these past studies, we found that, across treatments, ~5.6% of the root P content was from derived from QD-apatite (Fig. 4).

Although our results confirm that larger, established mycorrhizal roots directly adjacent to nutrient sources may initially dominate nutrient uptake [73], we found that this can change over time as the fungal network continues to relocate nutrients to other host roots. Past work has shown how seedlings receive nutrients from mycorrhizal networks, despite not being the main sinks [74–76]. However, it has also been shown that smaller plants can suffer when connected to larger plants via a mycorrhizal fungus [29]. A review of the literature found no clear pattern in how seedlings respond to being incorporated into mycorrhizal networks, with a meta-analysis finding that roughly half (42%) of the seedling species investigated responded positively to the presence of a CMN, while negative or neutral effects were found in the remaining cases [77]. We argue that this lack of consistency may be driven by a time component. Our work suggests that the transfer of P from the fungal network is highly dynamic and that nutrient allocation dynamics need to be measured across multiple time points, and potentially over even longer time scales than the 3 weeks measured here.

A second issue is the sink dynamics of the host roots. If the established roots had depleted their C source by week 3, then there is the question of whether they could still “pay” for their P. This could result in more P being allocated to the younger root simply because of C limitation in the established root. However, by adding excess sucrose to the MSR medium (10 gL^−1^), we ensured there was no C shortage. This was confirmed by our imaging data (Fig. S1), which demonstrated that root size increased at high rates, (between ~1 and 3 mm^2^/h, for the first ~3 months) with no evidence of C shortage to fungal allocation even after 5 months.

### Fungal nutrient allocation strategies: retention or transport

Using confocal microscopy, we were able confirm that the QD-apatite was taken up by the fungal hyphae and transferred from intraradical hyphae to the host roots (Fig. 5). Specifically, our confocal RICS technique allowed us to visually determine the proportion of QD-apatite physically retained in the hyphae versus transferred to the host roots.

Based on data from the first addition, we observed that the ratio of QD-apatite retained in the hyphae did not differ in established or young roots, or under control and low-P treatments (Fig. 5). This ratio of QD-apatite retained in hyphae divided by amount transferred to roots was slightly below one, suggesting that equal amounts of QD-apatite per biovolume were present in both tissue types. However, by the third injection, we saw a different pattern: a lower ratio indicated that more QD-apatite was transferred to the roots than retained in the hyphae. This ratio was significantly lower in the young roots under low-P conditions versus control conditions, suggesting low resource conditions stimulated more nutrient transfer to roots and less hyphal storage (Fig. 5). Because historically there has been an inability to separately quantify nutrients into those transferred to the roots and those retained in the hyphae, it has been difficult to study nutrient allocation strategies from a fungal perspective. This new resolution offers the potential to quantify these strategies over space and time using differently colored nutrients in combination with precision RICS techniques [38, 50, 51, 78, 79].

### Fungal benefit

We have shown that transfer of nutrients from fungal networks to hosts can be influenced by the host’s nutrient status: more QD-apatite was transferred to young roots under low-P conditions (Figs. 2 and 3). We suggest that the fungi employ this strategy not to “help hosts” as is often invoked in the literature, but rather because this strategy has the potential to increase direct benefits to the fungus. Theoretical [34] and empirical [24, 25] work suggests that, rather than a fixed exchanged of nutrients between partners, allocation of resources depends strongly on context, such as host growth conditions [23–26], externally available nutrients [19, 20, 80, 81] and number of competing fungi [82]. Specifically, biological market theory predicts that as a resource becomes more limiting, the value and hence price, of that resource potentially increases [34, 83, 84].

One of our aims was to test whether the fungi benefited from providing hosts growing under low-P conditions with an increasingly valuable resource. Specifically, we were interested in cumulative measures of C allocation by the host root to the symbiont. However, C allocation from host to arbuscular mycorrhizal fungi is notoriously difficult to measure. Physiological limitations will likely prevent the development of QD-tagged C because organisms consume C faster than P, potentially degrading QDs over time, exposing the heavy metal core of the QD, and leading to toxicity. Also, it is questionable as to whether QD-tagged C, for example, QD labeled hexose injected into root organ cultures, could ever serve as a relevant proxy for host C allocation dynamics. Traditional C labeling techniques likewise make it difficult to obtain accurate “cumulative” patterns as C is rapidly respired. However, new approaches using NanoSIMS (Nanoscale Secondary Ion Mass Spectrometry), and even NanoSIP (Nanoscale Stable Isotope Probing), may prove useful in tracking C pathways at the nanoscale in the future [85].

Given the limitations of cumulative C tracking, we measured the extraradical biomass of the fungi as a proxy for fungal benefit. Biomass can be a useful proxy because arbuscular mycorrhizal fungi are obligate biotrophs, meaning all their C is supplied directly from the host. Using the biomass measured from destructive harvesting of the samples, we did not find higher total biomass of extraradical hyphae in the low-P conditions (Fig. 6a). However, biomass measurements are imperfect because it is unknown how much C was allocated per each root system (i.e., established versus young) because C can be transferred across the fungal network. Therefore, we cannot exclude that C gained in the younger root compartment was transferred to the established root compartment, and vice versa. Ideally, we would use a method to quantify continuous C allocation that did not rely on destructive harvesting. New high-resolution imaging techniques, that convert the visual structures of mycorrhizal hyphal networks to biomass proxies, are a potential way forward.

### Open questions regarding QD-tagged nutrients

While we found support for our hypothesis that mycorrhizal networks transfer more P to the hosts with a higher nutrient demand, we still have many open questions regarding the QD-nutrient tagging technology. For example, it has not yet been possible to quantify the rate at which P is dissociated from the QD core across different biological tissue. One approach would be to use anisotropy techniques, which involve measuring the rotational speed of partly dissolved versus fully intact QD-apatite in different biological tissue. However, this requires that the QD-tagged nutrients have a fixed dipole moment, which is unlikely. A second approach is to create “radioactive QDs” to determine if the P from the nanoparticles becomes incorporated into DNA or phospholipids. However, the dual radioactive labeling + QD approach still fails to give a direct measurement of how much of the P remains attached to the QD. Rather, it can only tell us that the radioactive P initially attached to the QD core was eventually used in building biological tissue. Lastly, Raman microscopy is theoretically possible, although typical Raman signals are weak, and thus require long measurement times. Since Raman microscopy relies on monitoring vibrations, it will be necessary to find a specific vibration energy different enough from the vibration energies of other covalent bonds of other molecules in the cell. Therefore, it is an open question whether QD vibrations are both specific and strong enough to overcome the signals from the rest of the cell.

While exact decay measures of P dissolution from the QD tags are difficult to obtain, there is both direct and indirect evidence that QD-tagged nutrients are being used by the root cells to build biological material. For example, there is data showing that *Medicago* plants colonized by arbuscular mycorrhizal fungi grow equally well on apatite versus QD-tagged apatite when these nutrients are their sole P source. If the P from the QD-apatite was not available or even toxic, we would expect a growth depression in replicates grown on QD-apatite compared to apatite [38]. Furthermore, by measuring total P concentrations of roots versus P contribution from QD-apatite (Figs. 4 and S2), we show that host roots will use significantly more QD-apatite when grown in low-P conditions. This effect would not emerge if the tagged P was biologically unavailable. Lastly, we have direct evidence of QD-apatite being translocated in root cell compartment using RICS. Translocation from the fungus to roots cells is expected only when P remains conjugated to the QDs. Past work has shown that when fungal hyphae are exposed to unconjugated carboxyl terminated QDs, there is no uptake or translocation of QDs to the root: after 60 days of exposure values remained lower than the detection limit; <0.000001 nmol QD mg^−1^ plant tissue [38].

## Conclusion

Studying the nutrient allocation strategies of symbiotic microbes to their hosts remains a major challenge [84, 86]. In the case of arbuscular mycorrhizal fungi, slight alterations to the composition or growth conditions for the fungal network can result in shifts in nutrient trading strategies [87]. We have shown that time and host nutrient demand both play an important role in fungal allocation patterns. Under natural conditions, allocation patterns are likely to very dynamic and difficult to predict. This is especially true as host species composition changes, and abiotic factors vary seasonally [26, 29, 37, 88].

Utilizing QD techniques to study symbiotic trade is still in its infancy, but the technique can begin to be a useful tool to help us understand how fungal allocation patterns change under these various environmental contexts, across both space and time. Until we obtain exact QD-apatite decay measurements across different tissues, and we successfully characterize the QD-apatite uptake mechanisms by fungi and transfer from fungi to roots, caution is still needed. As these approaches become more refined, we can test predictions of resource exchange in host-symbiont relationships [34, 89], and determine the conditions under which partnerships are likely to bring direct and measurable benefits.

## Supplementary information

Supplementary Figure Legends

Video S1

Video S2

Figure S1

Figure S2

Figure S3

Table S1

## References

[1] Wipf D, Krajinski F, van Tuinen D, Recorbet G, Courty P (2019). Trading on the arbuscular mycorrhiza market: from arbuscules to common mycorrhizal networks. N Phytol.

[2] Miller RM, Jastrow JD, Reinhardt DR (1995). External hyphal production of vesicular-arbuscular mycorrhizal fungi in pasture and tallgrass prairie communities. Oecologia.

[3] Leake J, Johnson D, Donnelly D, Muckle G, Boddy L, Read DJ (2004). Networks of power and influence: the role of mycorrhizal mycelium in controlling plant communities and agroecosystem functioning. Can J Bot.

[4] Bago B, Pfeffer PE, Shachar-Hill Y (2000). Carbon metabolism and transport in arbuscular mycorrhizas. Plant Physiol.

[5] Drigo B, Pijl AS, Duyts H, Kielak AM, Gamper HA, Houtekamer MJ (2010). Shifting carbon flow from roots into associated microbial communities in response to elevated atmospheric CO2. Proc Natl Acad Sci.

[6] Giri B, Saxena B. Response of arbuscular mycorrhizal fungi to global climate change and their role in terrestrial ecosystem C and N cycling. In: Varma A, Prasad R, Tuteja N editors. Mycorrhiza—function, diversity, state of the art. Cham: Springer International Publishing; 2017. p. 305–27.

[7] Field KJ, Pressel S, Duckett JG, Rimington WR, Bidartondo MI (2015). Symbiotic options for the conquest of land. Trends Ecol Evol.

[8] Martin FM, Uroz S, Barker DG (2017). Ancestral alliances: plant mutualistic symbioses with fungi and bacteria. Science.

[9] Brundrett MC (2002). Coevolution of roots and mycorrhizas of land plants. N Phytol.

[10] Werner GDA, Cornelissen JHC, Cornwell WK, Soudzilovskaia NA, Kattge J, West SA (2018). Symbiont switching and alternative resource acquisition strategies drive mutualism breakdown. Proc Natl Acad Sci.

[11] Gange AC, Stagg PG, Ward LK (2002). Arbuscular mycorrhizal fungi affect phytophagous insect specialism. Ecol Lett.

[12] Koricheva J, Gange AC, Jones T (2009). Effects of mycorrhizal fungi on insect herbivores: a meta-analysis. Ecology.

[13] Hart MM, Reader RJ, Klironomos JN (2003). Plant coexistence mediated by arbuscular mycorrhizal fungi. Trends Ecol Evol.

[14] Hiiesalu I, Pärtel M, Davison J, Gerhold P, Metsis M, Moora M (2014). Species richness of arbuscular mycorrhizal fungi: associations with grassland plant richness and biomass. N Phytol.

[15] Gerz M, Bueno CG, Zobel M, Moora M (2016). Plant community mycorrhization in temperate forests and grasslands: relations with edaphic properties and plant diversity. J Veg Sci.

[16] He X, Critchley C, Bledsoe C (2003). Nitrogen transfer within and between plants through common mycorrhizal networks (CMNs). CRC Crit Rev Plant Sci.

[17] Smith, Sally E., and David J. Read. Mycorrhizal symbiosis. 3rd edn. (Academic press, London, 2008).

[18] Luginbuehl LH, Menard GN, Kurup S, Van Erp H, Radhakrishnan GV, Breakspear A (2017). Fatty acids in arbuscular mycorrhizal fungi are synthesized by the host plant. Science.

[19] Liu A, Hamel C, Hamilton RI, Ma BL, Smith DL (2000). Acquisition of Cu, Zn, Mn and Fe by mycorrhizal maize (Zea mays L.) grown in soil at different P and micronutrient levels. Mycorrhiza.

[20] Azcón R, Ambrosano E, Charest C (2003). Nutrient acquisition in mycorrhizal lettuce plants under different phosphorus and nitrogen concentration. Plant Sci.

[21] Ramírez-Viga TK, Aguilar R, Castillo-Argüero S, Chiappa-Carrara X, Guadarrama P, Ramos-Zapata J (2018). Wetland plant species improve performance when inoculated with arbuscular mycorrhizal fungi: a meta-analysis of experimental pot studies. Mycorrhiza.

[22] Weremijewicz J, Janos DP (2013). Common mycorrhizal networks amplify size inequality in *Andropogon gerardii* monocultures. N Phytol.

[23] Bücking H, Shachar-Hill Y (2005). Phosphate uptake, transport and transfer by the arbuscular mycorrhizal fungus Glomus intraradices is stimulated by increased carbohydrate availability. N Phytol.

[24] Fellbaum CR, Gachomo EW, Beesetty Y, Choudhari S, Strahan GD, Pfeffer PE (2012). Carbon availability triggers fungal nitrogen uptake and transport in arbuscular mycorrhizal symbiosis. Proc Natl Acad Sci.

[25] Fellbaum CR, Mensah JA, Cloos AJ, Strahan GE, Pfeffer PE, Kiers ET (2014). Fungal nutrient allocation in common mycorrhizal networks is regulated by the carbon source strength of individual host plants. N Phytol.

[26] Konvalinková T, Püschel D, Janoušková M, Gryndler M, Jansa J (2015). Duration and intensity of shade differentially affects mycorrhizal growth- and phosphorus uptake responses of *Medicago truncatula*. Front Plant Sci.

[27] Zheng C, Ji B, Zhang J, Zhang F, Bever JD (2015). Shading decreases plant carbon preferential allocation towards the most beneficial mycorrhizal mutualist. N Phytol.

[28] Varga S, Kytöviita M (2010). Mycorrhizal benefit differs among the sexes in a gynodioecious species. Ecology.

[29] Merrild MP, Ambus P, Rosendahl S, Jakobsen I (2013). Common arbuscular mycorrhizal networks amplify competition for phosphorus between seedlings and established plants. N Phytol.

[30] Walder F, Brulé D, Koegel S, Wiemken A, Boller T, Courty PE (2015). Plant phosphorus acquisition in a common mycorrhizal network: regulation of phosphate transporter genes of the Pht1 family in sorghum and flax. N Phytol.

[31] Weremijewicz J, Sternberg L, da SLO, Janos DP (2016). Common mycorrhizal networks amplify competition by preferential mineral nutrient allocation to large host plants. N Phytol.

[32] Werner GDA, Kiers ET (2015). Partner selection in the mycorrhizal mutualism. N Phytol.

[33] Bachelot B, Lee CT (2018). Dynamic preferential allocation to arbuscular mycorrhizal fungi explains fungal succession and coexistence. Ecology.

[34] Wyatt GAK, Kiers ET, Gardner A, West SA (2014). A biological market analysis of the plant-mycorrhizal symbiosis. Evolution.

[35] Noë R, Kiers ET (2018). Mycorrhizal markets, firms, and co-ops. Trends Ecol Evol.

[36] Bender SF, Wagg C, van der Heijden MGA (2016). An underground revolution: biodiversity and soil ecological engineering for agricultural sustainability. Trends Ecol Evol.

[37] Konvalinková T, Jansa J (2016). Lights off for arbuscular mycorrhiza: on its symbiotic functioning under light deprivation. Front Plant Sci.

[38] Whiteside MD, Werner GDAA, Caldas VEA, van’t Padje A, Dupin SE, Elbers B (2019). Mycorrhizal fungi respond to resource inequality by moving phosphorus from rich to poor patches across networks. Curr Biol.

[39] Bailey RE, Nie S (2003). Alloyed semiconductor quantum dots: tuning the optical properties without changing the particle size. J Am Chem Soc.

[40] Jang E, Jun S, Pu L. High quality CdSeS nanocrystals synthesized by facile single injection process and their electroluminescence. Chem Commun. 2003;24:2964–5. 10.1039/b310853h 14703809

[41] Declerck S, Fortin JA, Strullu DG (eds). In vitro culture of mycorrhizas. Berlin, Heidelberg: Springer; 2005.

[42] Engelmoer DJP, Behm JE, Kiers ET (2014). Intense competition between arbuscular mycorrhizal mutualists in an in vitro root microbiome negatively affects total fungal abundance. Mol Ecol.

[43] Ness RLL, Vlek PLG (2000). Mechanism of calcium and phosphate release from hydroxy-apatite by mycorrhizal hyphae. Soil Sci Soc Am J.

[44] Tang I-M, Krishnamra N, Charoenphandhu N, Hoonsawat R, Pon-On W (2010). Biomagnetic of apatite-coated cobalt ferrite: a core–shell particle for protein adsorption and pH-controlled release. Nanoscale Res Lett.

[45] Kawashita M, Taninai K, Li Z, Ishikawa K, Yoshida Y (2012). Preparation of low-crystalline apatite nanoparticles and their coating onto quartz substrates. J Mater Sci Mater Med.

[46] Sun S, Chan LS, Li Y-L (2014). Flower-like apatite recording microbial processes through deep geological time and its implication to the search for mineral records of life on Mars. Am Miner.

[47] Kiers ET, Duhamel M, Beesetty Y, Mensah JA, Franken O, Verbruggen E (2011). Reciprocal rewards stabilize cooperation in the mycorrhizal symbiosis. Science.

[48] R core team. R: a language and environment for statistical computing. Vienna: R Foundation for Statistical Computing; 2018. https://www.r-project.org/.

[49] Walker C (2005). A simple blue staining technique for arbuscular mycorrhizal and other root-inhabiting fung. Inoculum.

[50] Rossow MJ, Sasaki JM, Digman MA, Gratton E (2010). Raster image correlation spectroscopy in live cells. Nat Protoc.

[51] Whiteside MD, Digman MA, Gratton E, Treseder KK (2012). Organic nitrogen uptake by arbuscular mycorrhizal fungi in a boreal forest. Soil Biol Biochem.

[52] Bates D, Mächler M, Bolker B, Walker S. “Fitting Linear Mixed-Effects Models Using lme4.” Journal of Statistical Software. 2015. 67;1:1–48.

[53] Kuznetsova A, Brockhoff PB, Christensen RHB (2017). “lmerTest Package: Tests in Linear Mixed Effects Models.” Journal of Statistical Software. 2017. 82;13:1–26.

[54] Fox J, Weisberg S. An R companion to applied regression. 2nd edn (Sage Publications, Inc, Thousand Oaks CA, 2016).

[55] Javot H, Pumplin N, Harrison MJ (2007). Phosphate in the arbuscular mycorrhizal symbiosis: transport properties and regulatory roles. Plant Cell Environ.

[56] Konečný J, Hršelová H, Bukovská P, Hujslová M, Jansa J (2019). Correlative evidence for co-regulation of phosphorus and carbon exchanges with symbiotic fungus in the arbuscular mycorrhizal *Medicago truncatula*. PLoS ONE.

[57] Keymer A, Pimprikar P, Wewer V, Huber C, Brands M, Bucerius SL (2017). Lipid transfer from plants to arbuscular mycorrhiza fungi. Elife.

[58] Burleigh SH, Cavagnaro T, Jakobsen I (2002). Functional diversity of arbuscular mycorrhizas extends to the expression of plant genes involved in P nutrition. J Exp Bot.

[59] Smith SE (2003). Mycorrhizal fungi can dominate phosphate supply to plants irrespective of growth responses. Plant Physiol.

[60] Grønlund M, Albrechtsen M, Johansen IE, Hammer EC, Nielsen TH, Jakobsen I (2013). The interplay between P uptake pathways in mycorrhizal peas: a combined physiological and gene-silencing approach. Physiol Plant.

[61] Smith SE, Smith FA, Jakobsen I (2004). Functional diversity in arbuscular mycorrhizal (AM) symbioses: the contribution of the mycorrhizal P uptake pathway is not correlated with mycorrhizal responses in growth or total P uptake. N Phytol.

[62] Watts-Williams SJ, Jakobsen I, Cavagnaro TR, Grønlund M (2015). Local and distal effects of arbuscular mycorrhizal colonization on direct pathway Pi uptake and root growth in *Medicago truncatula*. J Exp Bot.

[63] Pel R, Dupin S, Schat H, Ellers J, Kiers ET, van Straalen NM (2018). Growth benefits provided by different arbuscular mycorrhizal fungi to *Plantago lanceolata* depend on the form of available phosphorus. Eur J Soil Biol.

[64] Reynolds HL, Vogelsang KM, Hartley AE, Bever JD, Schultz PA (2006). Variable responses of old-field perennials to arbuscular mycorrhizal fungi and phosphorus source. Oecologia.

[65] Lu R, Drubin DG, Sun Y (2016). Clathrin-mediated endocytosis in budding yeast at a glance. J Cell Sci.

[66] Fischer-Parton S, Parton RM, Hickey PC, Dijksterhuis J, Atkinson HA, Read ND (2000). Confocal microscopy of FM4-64 as a tool for analysing endocytosis and vesicle trafficking in living fungal hyphae. J Microsc.

[67] Read ND, Kalkman ER (2003). Does endocytosis occur in fungal hyphae?. Fungal Genet Biol.

[68] Epp E, Nazarova E, Regan H, Douglas LM, Konopka JB, Vogel J (2013). Clathrin- and arp2/3-independent endocytosis in the fungal pathogen *Candida albicans*. MBio.

[69] Colin Y, Nicolitch O, Turpault MP, Uroz S (2017). Mineral types and tree species determine the functional and taxonomic structures of forest soil bacterial communities. Appl Environ Microbiol.

[70] Fontaine L, Thiffault N, Paré D, Fortin J-A, Piché Y (2016). Phosphate-solubilizing bacteria isolated from ectomycorrhizal mycelium of Picea glauca are highly efficient at fluorapatite weathering. Botany.

[71] Alloush GA, Clark RB (2001). Maize response to phosphate rock and arbuscular mycorrhizal fungi in acidic soil. Commun Soil Sci Plant Anal.

[72] Powell CL, Daniel J (1978). Mycorrhizal fungi stimulate uptake of soluble and insoluble phosphate fertilizer from a phosphate‐deficient soil. N Phytol.

[73] Jakobsen I, Hammer EC. Nutrient dynamics in arbuscular mycorrhizal networks. In: Horton TR, editor. Mycorrhizal networks. Dordrecht: Springer Netherlands; 2015. p. 91–131.

[74] Marler MJ, Zabinski CA, Callaway RM (1999). Mycorrhizae indirectly enhance competitive effects of an invasive forb on a native bunchgrass. Ecology.

[75] Carey EV, Marler MJ, Callaway RM (2004). Mycorrhizae transfer carbon from a native grass to an invasive weed: evidence from stable isotopes and physiology. Plant Ecol.

[76] van der Heijden MGA (2004). Arbuscular mycorrhizal fungi as support systems for seedling establishment in grassland. Ecol Lett.

[77] van der Heijden MGA, Horton TR (2009). Socialism in soil? The importance of mycorrhizal fungal networks for facilitation in natural ecosystems. J Ecol.

[78] Digman MA, Brown CM, Sengupta P, Wiseman PW, Horwitz AR, Gratton E (2005). Measuring fast dynamics in solutions and cells with a laser scanning microscope. Biophys J.

[79] Nieves DJ, Li Y, Fernig DG, Levy R (2015). Photothermal raster image correlation spectroscopy of gold nanoparticles in solution and on live cells. R Soc Open Sci.

[80] Johnson NC, Graham JH, Smith FA (1997). Functioning of mycorrhizal associations along the mutualism-parasitism continuum. N Phytol.

[81] Johnson NC, Wilson JA, Bowker MA, Wilson JA, Miller RM (2010). Resource limitation is a driver of local adaptation in mycorrhizal symbioses. Proc Natl Acad Sci.

[82] Argüello A, O’Brien MJ, van der Heijden MGA, Wiemken A, Schmid B, Niklaus PA (2016). Options of partners improve carbon for phosphorus trade in the arbuscular mycorrhizal mutualism. Ecol Lett.

[83] Noë R, Hammerstein P (1994). Biological markets: supply and demand determine the effect of partner choice in cooperation, mutualism and mating. Behav Ecol Sociobiol.

[84] Werner GDA, Strassmann JE, Ivens ABF, Engelmoer DJP, Verbruggen E, Queller DC (2014). Evolution of microbial markets. Proc Natl Acad Sci.

[85] Musat N, Musat F, Weber PK, Pett-Ridge J (2016). Tracking microbial interactions with NanoSIMS. Curr Opin Biotechnol.

[86] Bücking H, Mensah JA, Fellbaum CR (2016). Common mycorrhizal networks and their effect on the bargaining power of the fungal partner in the arbuscular mycorrhizal symbiosis. Commun Integr Biol.

[87] Roger A, Colard A, Angelard C, Sanders IR (2013). Relatedness among arbuscular mycorrhizal fungi drives plant growth and intraspecific fungal coexistence. ISME J.

[88] Wagg C, Jansa J, Schmid B, van der Heijden MGA (2011). Belowground biodiversity effects of plant symbionts support aboveground productivity. Ecol Lett.

[89] Douglas AE (2008). Conflict, cheats and the persistence of symbioses. N Phytol.

