# Supplementary material for: Temporal tracking of quantum-dot apatite across in vitro mycorrhizal networks shows how host demand can influence fungal nutrient transfer strategies
Source: ISME J. 2020 Sep 28;15(2):435–49. doi: 10.1038/s41396-020-00786-w (PMC8027207; doi:10.1038/s41396-020-00786-w)
Supplement: Supplementary file 1 — Supplementary Figure Legends [file 41396_2020_786_MOESM1_ESM.docx]

**Supplementary Figure Legends**

**Figure S1. Root growth rate over time.** **(a)** Surface area occupied by root over time in six host root replicates. The symbols correspond to the inoculated (gray-squares) and non-inoculated (black-circles) root. The dashed-lines serves as a visual guide. **(b)** A zoomed-in image sequence of an inoculated root is shown. After five months, the growth and sporulation of the extraradical fungal network is still observable. The white arrows point to single spores or clusters. The thin mycelium connecting the spores correspond to the fungal network. (n_inoculated_=3, n_non-inoculated_=3).

**Figure S2. P concentration in host roots.** (**a**) Total P concentration in nmol/mg host roots. P concentration was significantly higher in the control roots compared to roots growing under low-P conditions. The asterisk indicates a p-value < 0.05. Means ± SEM.

**Figure S3.** **Arbuscular mycorrhizal colonization of host roots per treatment per compartment.** We confirmed the presence of hyphal colonization by microscopy. **(a)** The percentage of hyphal colonization. **(b)** The percentage of arbuscules. **(c)** The percentage of vesicles. (n_control,established_=5, n_control,young_ =2, n_low-P,established_ =5, n_low-P,young_=1).

**Video S1. Synchronous Bright-field and Fluorescence video from a transformed root organ culture inoculated with *R. irregularis* exposed to QD-apatite**. A week after the addition of the QD-apatite solution, clogging is induced in the intrahyphal flow due to formation of large vacuoles inside the hyphae. The synchronous illumination allows to observe the QD-apatite (big-bright areas) and the cytoplasmic flow at the same time. The video has color ascribed via Lookup table (LUT) using imageJ (mpl-plasma color palette).

**Video S2. Bright-field video from a transformed root organ culture inoculated with *R. irregularis* not exposed to QD-apatite**. A simultaneous bi-directional flow is observed over the time, towards and away the root. In this case, when no QD-apatite is added, no translocation of large vacuoles is observed. The video has color ascribed via Lookup table (LUT) using imageJ (mpl-plasma color palette).

**Table S1.** Summary of the data, number of replicates (n), mean, standard deviation (SD), standard error or the mean (SE) and 95% confidence intervals (CI) are provided.
