# Supplementary figures and images for: Temporal tracking of quantum-dot apatite across in vitro mycorrhizal networks shows how host demand can influence fungal nutrient transfer strategies
Source: ISME J. 2020 Sep 28;15(2):435–49. doi: 10.1038/s41396-020-00786-w (PMC8027207; doi:10.1038/s41396-020-00786-w)

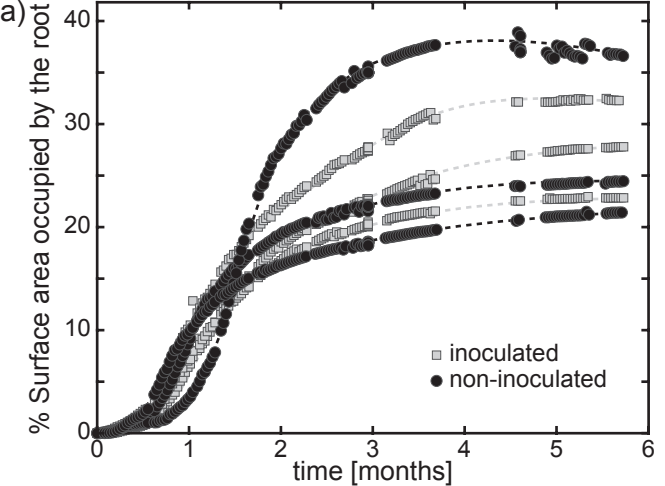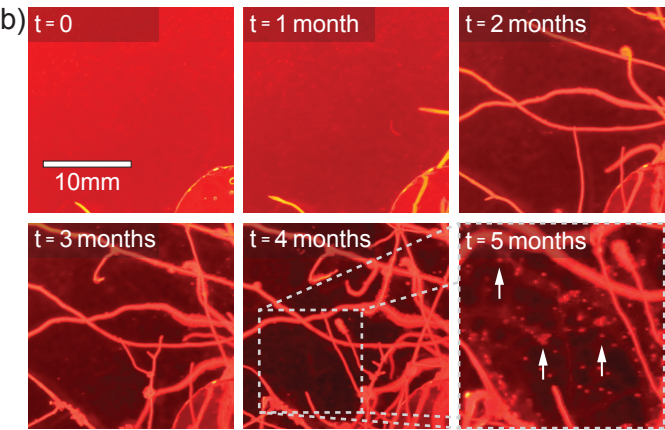

Supplement: Supplementary file 4 — Figure S1 [file 41396_2020_786_MOESM4_ESM.pdf]

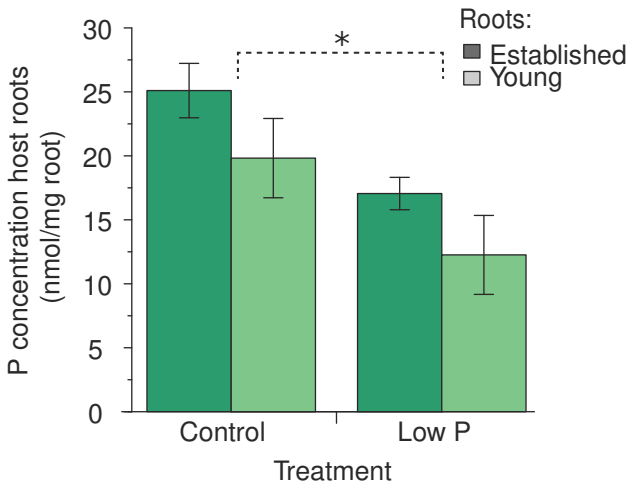

Supplement: Supplementary file 5 — Figure S2 [file 41396_2020_786_MOESM5_ESM.pdf]

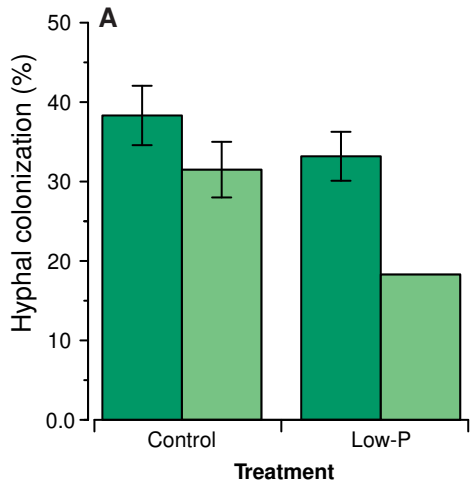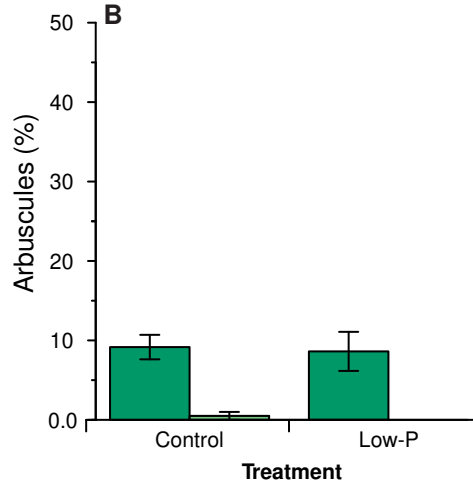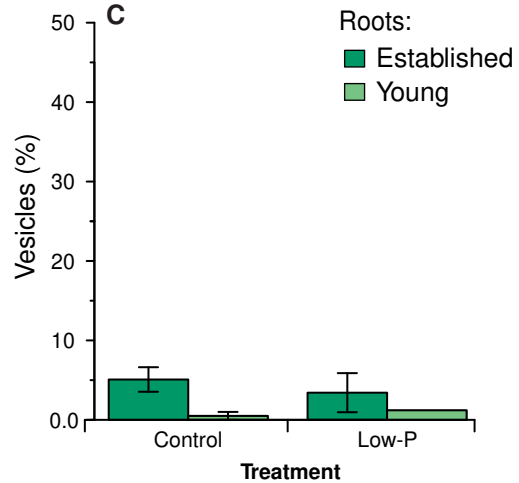

Supplement: Supplementary file 6 — Figure S3 [file 41396_2020_786_MOESM6_ESM.pdf]
